# Editorial: Reviews in gastrointestinal cancers

**DOI:** 10.3389/fonc.2023.1252665

**Published:** 2023-07-18

**Authors:** Kanjoormana Aryan Manu, Fabrizio Bronte, Emilio Francesco Giunta

**Affiliations:** ^1^ Department of Immunology, Amala Cancer Research Centre, Thrissur, Kerala, India; ^2^ Gastroenterology Unit, Department of Medicine, Ospedali Riuniti Villa Sofia- V. Cervello, Palermo, Italy; ^3^ Department of Experimental Medicine, Università degli Studi della Campania “Luigi Vanvitelli”, Naples, Italy

**Keywords:** gastrointestnal cancers, gastric cancer, liver cancer, pancreatic cancer, colorectal cancer, cancer diagnosis, cancer treatment

Gastrointestinal (GI) cancers are a group of cancers associated with the gastrointestinal tract and the most affected areas are the esophagus, stomach, colon, liver, and pancreas. GI cancers are responsible for 25% of cancer incidence and 33% of cancer-related death globally. Even though the recent advances in diagnosis and therapies have made an overall good impact, the challenges in controlling and managing GI cancers continue (1). This Research Topic with 34 articles is intended to consolidate the present status of research in the diagnosis and treatment of GI cancers.

The world is now witnessing the emergence of artificial intelligence (AI) in every field. Luo et al. assessed that AI is highly accurate in early-stage upper GI cancer detection using endoscopic images. In a systematic review, Jia et al. showed by metanalysis that AI Deep learning models have higher predictive accuracy than radiomics models in patients with rectal cancer.

Arrichiello et al. describe the emerging pathological features to predict the prognosis of patients with colorectal cancer (CRC). In a systematic review by Guan et al., the authors have performed a meta-analysis to quantify the relevance of preoperative factors for peritoneal carcinomatosis in gastric cancer when using staging laparoscopy (SL). He et al., Schlosser et al., and Sung et al. have reviewed the emerging biomarkers and their potential for clinical diagnosis of hepatocellular carcinoma (HCC). In a systematic review and meta-analysis, Yang et al. assessed the prognostic value of pan-immune-inflammation value in patients with CRC. In another systematic review, Li et al. assessed the diagnostic value of lncRNAs for gastric cancer.

In a case report, Zhang et al. described a condition of pancreatic neuroendocrine tumors and liver perivascular tumors with the involvement of multiple organs and space-occupying lesions, which is less common. Yan et al. summarized that interventional therapies such as rupture tissue ablation and TAE/TACE for those who are not tolerant to emergency surgery, reach an ideal prognosis for ruptured HCC (rHCC) cases. Xue et al. reviewed the prognostic importance of tumor budding, which is a single cell or cluster of up to four cells at the cancer invasion margin in gastric cancer. Bae et al. showed an increasing trend in the utilization of radiotherapy, adoption of advanced techniques, and overall survival improvements in patients with HCC from a Korean tertiary hospital registry. A review by Wang Q et al. has described the pathogenesis, diagnosis, and management of an extremely rare pathological condition, primary hepatopancreatobiliary lymphoma, and offers a diagnosis and management schedule for clinicians. Chen et al. have reported a meta-analysis on neoadjuvant chemoradiotherapy for resectable gastric cancer. Li et al. have discussed the current status and future perspectives of cardia preserving radical gastrectomy, which is a promising approach with various advantages. Du et al. have reported a rare case of an ectopic enterogenous cyst in the anterior sacral and soft tissue of the buttocks and its carcinomatous transformation, an event that has never been reported before in the literature.

Comparative efficacy and toxicity of immune checkpoint inhibitors (ICIs) combined or not with chemotherapy have been analyzed in the systematic review by Ma et al.. Wu et al. updated the advancement in the research of HCC progression after radiofrequency ablation. In a systematic review, Zhong et al. assessed the efficacy and safety of ICIs combined with antiangiogenic drugs in HCC. Hu et al. described the current advances in research on the secondary resistance to imatinib against gastrointestinal stromal tumors (GISTs). A meta-analysis by Zheng et al. showed the effect of phosphoglucomutase (PGM), a key enzyme involved in the synthesis and breakdown of glycogen, on the survival prognosis of tumor patients. In this Research Topic, Jiang et al. have discussed the possibilities of targeting neutrophils against the development and progression of pancreatic cancer.

Qiu et al. reviewed the role of Intercellular Adhesion Molecule-1 (ICAM-1), a cell surface glycoprotein, focusing on expression, functions, prognosis, tumorigenesis, polymorphism, and therapeutic implications in CRCs. Gong et al. have reviewed the role of melatonin, a natural indolamine in inhibiting GI carcinogenesis and the mechanisms behind it. Xie et al. have reviewed the application of single-cell sequencing in gastrointestinal cancers. Qi et al. have described the prognostic roles of Competitive Endogenous RNAs (ceRNA) Netowork-Based Signatures in GI cancers. Wang S. et al. have reviewed the mechanisms and prospects of circular RNAs, a group of single-stranded RNAs that form a covalently closed continuous loop, and their role in GI cancer signaling networks.

Up to 80% of patients with pancreatic adenocarcinoma (PDAC) can experience PDAC-derived cachexia, a systemic disease that involves a complex interplay between the tumor and multiple organs. Yu et al. reviewed the endocrine organ-like tumor hypothesis that explains the factors involved in cachexia development. Ferroptosis is an iron-dependent form of programmed cell death and Liang et al. have reviewed the therapeutic applications of ferroptosis in CRCs. In their review, Melia et al. have explained the pro-tumorigenic role of type-2 diabetes-induced cellular senescence in CRCs and its molecular mechanism. In a systematic review, Pang et al. investigated the clinical significance of lung cancer inflammation index in patients with GI cancer in order to evaluate the postoperative complications before surgery and survival outcomes.

Taken together, the articles published in this Research Topic have discussed a broad area of research in GI cancers, from diagnosis to therapeutic resistance. Current advances in genomic techniques and AI have certainly created new and tremendous possibilities in GI diagnosis and therapy, which will hopefully improve GI cancer management in the future.

## Author contributions

KM has written, FB and EG have reviewed the editorial. All authors contributed to the article and approved the submitted version.
